# Evaluating Signs of Pulmonary Hypertension on Computed Tomography and Correlating With Echocardiography: A Study at a Tertiary Care Hospital

**DOI:** 10.7759/cureus.25319

**Published:** 2022-05-25

**Authors:** Abdur Rehman, Jaideep Darira, Muhammad Saad Ahmed, Kamran Hamid, Muhammad Kashif Shazlee, Syed Muhammad Shahnawaz Hyder

**Affiliations:** 1 Radiology, Dr. Ziauddin Hospital, Karachi, PAK; 2 Diagnostic Radiology, Dr. Ziauddin Hospital, Karachi, PAK; 3 Radiology, Dr. Ziauddin University Hospital, Karachi, PAK

**Keywords:** main pulmonary artery to aorta ratio, main pulmonary artery, 2d echocardiography, computed tomography (ct ), – pulmonary hypertension

## Abstract

Introduction: Pulmonary hypertension (PH) is a threatening condition, and it is far more common than previously assumed, especially after the COVID pandemic. Its outcome is not good; if detected late, and can lead to right ventricular failure, which can be fatal. Our goal was to evaluate CT signs of PH, correlate them with echocardiography, and identify the cut-off values of these signs in our population.

Method: In this study, 160 patients having both CT and echocardiography with a maximum gap of one month were assessed from June to November 2021. The association between CT signs and echocardiography to diagnose PH was investigated. The Pearson and Spearman correlation and area under receiver operating curve (AUROC) tests were performed in the analysis. Receiver operating characteristic curve analysis was also used to assess CT’s diagnostic capability and cut-off values.

Result: The correlation between main pulmonary artery (MPA) diameter and main pulmonary artery to aorta ratio (MPA/AO) with mean pulmonary artery pressure (mPAP) was weak but statistically significant (r = 0.316 and r = 0.321, p<0.001). However, there was a very weak correlation between the right and left pulmonary artery and mPAP with correlation coefficients (r) of 0.155 and 0.138, respectively. For the first time in our population, we measured the cut-off values of MPA and MPA/AO ratios for PH which were 26 and 0.88 mm, respectively.

Conclusions: The CT signs of PH correlate with echocardiography; however, should not be used solely; the cut-off values should be used according to race and population.

## Introduction

Pulmonary hypertension (PH) is a complex illness that nearly invariably shortens life expectancy. Therapy is difficult, but getting a prompt diagnosis is also challenging [1]. It is stated that the mean pulmonary artery pressure (mPAP) is more than 25 mmHg in the resting state, assessed by cardiac catheterization [2]. It is one percent prevalent in the population of all ages. However, the prevalence increases significantly in people older than 65 [3]. In the recent pandemic of coronavirus disease 2019 (COVID-19), there is a risk that extensive COVID disease among affected patients might cause lasting harm to the lung parenchyma and vessels, favoring the establishment of PH in the future [4]. The prognosis for PH is poor; if not recognized and managed soon enough, this could proceed to right ventricular failure, which has a significant fatality [5]. Unexplained tiredness, exertional dyspnea, angina, and dizziness are some of the initial clinical symptoms of PH. Despite increasing recognition of PH, most cases still have delayed detection [6]. The gold standard for detecting PH is right heart catheterization by measuring mean pulmonary arterial systolic pressure (PASP) [7]. However, due to the invasiveness of the procedure, expense, or unavailability, many institutions across the globe do not use this procedure [8]. Non-invasive imaging methods are useful for detecting the existence of PH [9]. Echocardiography is indicated as the first step [10]. It is less invasive and widely available; it is now a significant part of the PH diagnosis process [11]. CT, either plain or contrast, is usually suggested and done in individuals with dyspnea, tiredness, or tachycardia. In these cases, CT could be the first method to indicate the diagnosis of PH in this situation.

Echocardiography measures the mPAP, size, thickness, and right ventricle function. On the other hand, CT shows the anatomy of the pulmonary arterial tree. There are variations in the cut-off values of CT parameters for PH within populations [12]. Ethnicity, patient factors, and variations in CT techniques might all contribute to the variance [13]. So we evaluated these signs in the population of Pakistan, correlated them with echocardiography, and predicted the cut-off values in our population for the first time.

## Materials and methods

This study was carried out exclusively at Dr. Ziauddin Hospital, Karachi, from June 2021 to November 2021. Since no human interaction was needed, a waiver was granted from the Ethical Review Committee of Dr. Ziauddin Hospital with Reference Number: 3630421ARRAD. Non-probability convenient sampling was used. Data were collected from 160 outpatients and inpatients of the hospital. The inclusion criterion included data from patients above 18 years who have undergone chest CT and echocardiography with a maximum one-month gap between the two. Chest CT and echocardiography with technical errors/difficulties were excluded.

Echocardiography was performed by XARIO 100 (Canon Medical Systems Corporation, Kawasaki, Japan) on patients included in this study. The mPAP was calculated by tricuspid regurgitation (TR) peak velocity. The following formula was used to calculate the PASP:

PASP = 4 (peak TR velocity)2 + RAP [right arterial pressure which was assessed by the diameter of inferior vena cava (IVC)]

Then PASP was converted into mPAP by using the following formula: 0.61 × PASP + 2 mmHg [14].

The SEIMENS multislice CT (Siemens AG Medical Solutions, Erlangen, Germany) scanner was used, and all of the patients were subjected to either a high-resolution computed tomography (HRCT) or a computed tomography pulmonary angiogram (CTPA). The CT parameters for PH were assessed as follows:

Diameter of the main pulmonary artery

The maximum diameter of MPA was calculated at the bifurcation point [15].

Main pulmonary artery diameter to ascending aorta ratio

The MPA/AO was measured on an axial picture at the right pulmonary artery bifurcation [16].

Right pulmonary artery and left pulmonary artery diameters

Following MPA bifurcation, the broadest section measurements of the right and left pulmonary arteries were taken [16].

Statistical analysis

The Statistical Package for the Social Sciences (SPSS) version 21 (IBM Inc., Armonk, NY) analyzed the data. The correlations between vessel measures (MPA, MPA/AO, RPA, and LPA) and vessel pressure (mPAP) were calculated using Pearson and Spearman correlations.

The p-value, sensitivity, and specificity of the collected data were determined. The cut-off value for the MPA and MPA/AO indicating PH was determined using a ROC curve, which indicates the diagnostic test’s precision. In order to measure the test’s diagnostic accuracy, the area under the curve was also determined.

## Results

One hundred and sixty cases were investigated for the study from June 2021 to November 2021. There were 72 females (45%) and 88 males in the group (55%). Patients ranged in age from 18 to 89 years old. mPAP was more than 25 mmHg in 118 patients (73.75%).

Correlation between main pulmonary artery and mean pulmonary artery pressure

The MPA diameter and mPAP values were observed to have a weak but statistically significant relationship where the value of r was 0.316 (p = 0.000), as shown in Table 1.

**Table 1:** Correlation between MPA and mPAP. mPAP, mean pulmonary artery pressure; MPA, main pulmonary artery *Correlation is significant at the 0.01 level (two-tailed).

		mPAP	MPA
mPAP	Pearson correlation	1	0.316^*^
	Sig. (two-tailed)		0.000
	N	160	160

Diagnostic performance of main pulmonary artery

**Figure 1:**
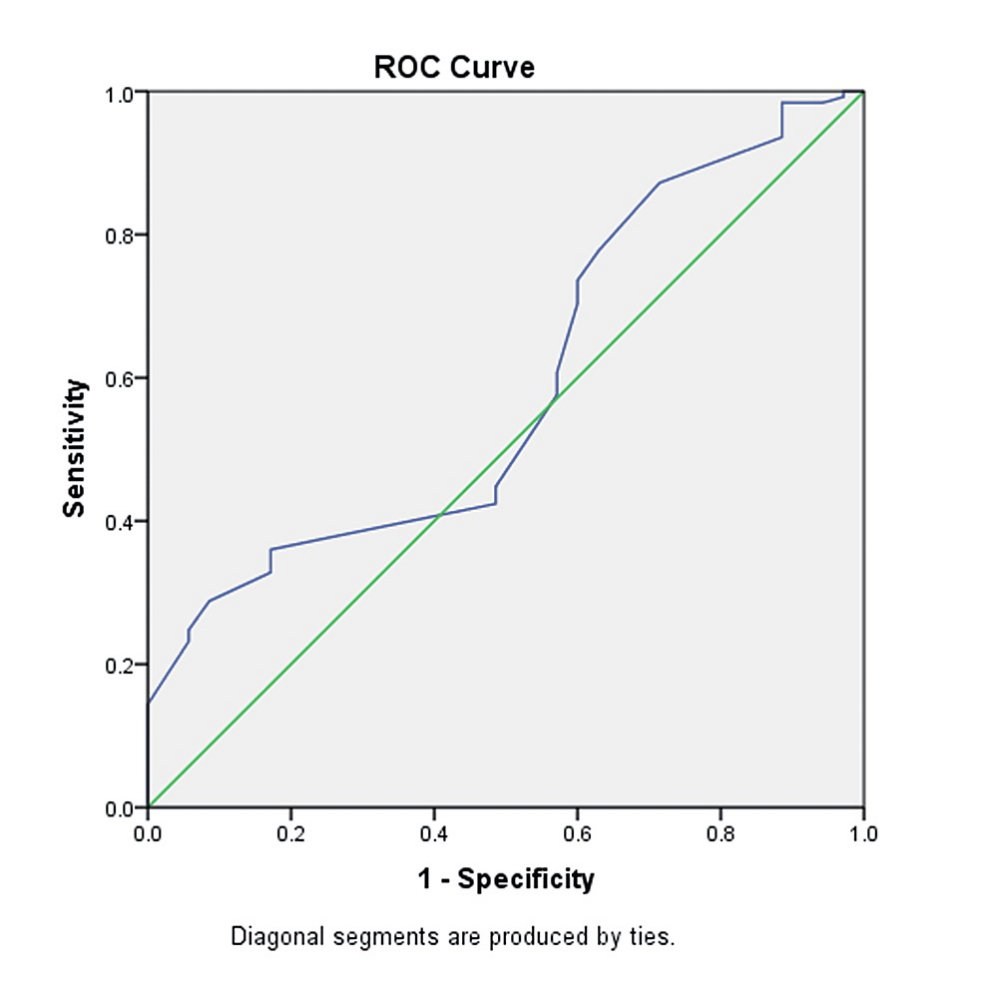
The ROC curve of MPA. MPA, mean pulmonary artery; ROC, receiver operating curve

Figure 1 is the graphical presentation of the ROC curve of analysis of MPA, as determined with the help of mPAP. The value of AUROC stood at 0.591. The sensitivity, specificity, and cut-off values were 0.608, 0.53, and 26 mm.

Correlation between main pulmonary artery to ascending aorta ratio (MPA/AO ratio) and mean pulmonary artery pressure

The MPAP and MPA/AO ratio were observed to have a weak but statistically significant relationship where the value of r was 0.321 (p = 0.000), as shown in Table 2.

**Table 2:** Correlation between MPA/AO ratio and mPAP. MPA/AO, main pulmonary artery to ascending aorta ratio; mPAP, mean pulmonary artery pressure. *Correlation is significant at the 0.01 level (two-tailed).

		mPAP	MPA/AO ratio
mPAP	Pearson correlation	1	0.321*
	Sig. (two-tailed)		0.000
	N	160	160

Diagnostic performance of main pulmonary artery to ascending aorta ratio

**Figure 2:**
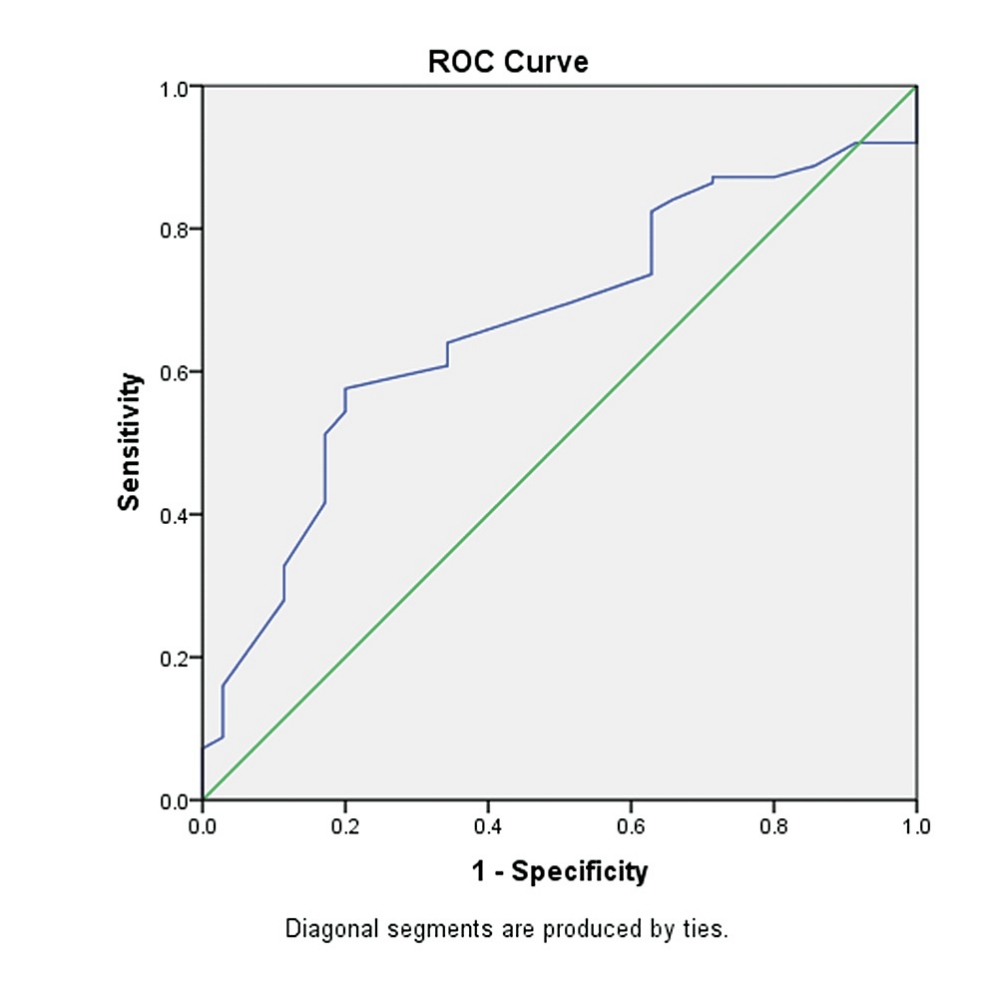
The ROC curve of MPA/AO ratio. ROC, receiver operating curve; MPA/AO, main pulmonary artery to aorta ratio

Figure 2 is the graphical presentation of the ROC curve of analysis of MPA/AO, as determined with the help of mPAP. The value of AUROC stands at 0.666. The sensitivity, specificity, and cut-off values are 0.640, 0.657, and 0.88, respectively.

Correlation between right and left pulmonary artery and mean pulmonary artery pressure

The RPA and LPA have a very weak association with MPAP (r=0.155 and 0.138, p-value=0.05 and 0.081, respectively), as shown in Tables 3-4. The correlation was not even significant in the case of the LPA.

**Table 3:** Correlation between RPA and mPAP. RPA, right pulmonary artery; mPAP, mean pulmonary artery pressure

			mPAP	RPA
Spearman's rho	mPAP	Correlation coefficient	1.000	0.155
		Sig. (two-tailed)	.	0.050
		N	160	160

 

**Table 4:** Correlation between LPA and mPAP. LPA, left pulmonary artery; mPAP, mean pulmonary artery pressure

			mPAP	LPA
Spearman's rho	mPAP	Correlation coefficient	1.000	0.138
		Sig. (two-tailed)	.	0.081
		N	160	160

## Discussion

Pulmonary hypertension presents a challenge for physicians as there are a number of potential underlying factors, each with its own management. CT can be quite helpful in determining the underlying causes of cardiac, vascular, and pulmonary problems. The prognosis of PH can be considerably improved if it is detected early. If left untreated, PH can lead to right heart failure and death [17].

This study revealed a weak (r = 0.316, p-value = 0.000), but statistically significant association between MPA diameter and mPAP. In research by Corson et al., the correlation coefficients among vascular measures and MPAP were also weak (r = 0.34) but significant for pulmonary hypertensive and non-pulmonary hypertensive patients [18]. In a study of 117 interstitial lung disease (ILD) patients, Fakhrain et al. also found a weak connection between MPA and mPAP (r=0.15, p-value=0.17) [19]. Moreover, Tan et al. [20] investigated 36 individuals who had right heart catheterization -- confirmed PH, and found no link between MPA size and mPAP. This absence of association was due to parenchymal lung disease and architectural distortion. In patients with fibrotic pulmonary disease, Zisman et al. and Devaraj et al. [21-22] also found no significant association between the MPA size and mPAP.

On the contrary, Mahammedi found moderate and statistically significant associations in a population of 298 PH patients and 102 non-PH patients, with r=0.51 and r=0.53, respectively [23]. There was also a moderate association between mPAP and MPA in both the ILD (r=0.608, p=0.001) and non-ILD cohorts (r=0.426, p=0.001) as researched by Chin et al. [24].

The measurement of pulmonary arterial pressure in individuals with PH depends on the pressure as well as on the period of the condition explaining the varying association measured between pulmonary arterial size and pressure in individuals with a diagnosis of PH [24].

The MPA/AO was weakly associated (r = 0.321, p-value=0.000) with MPAP. Corson et al. also had a weak correlation of r = 0.40 [18]. However, the study of Iyer et al. on 60 patients with chronic obstructive pulmonary disease (COPD) and Mahammadi et al. on 298 patients revealed moderate correlation (r = 0.56, r = 54 respectively) [23, 25]. Iyer et al. also showed that in individuals with severe COPD, the CT scan was shown to be more accurate than an echocardiogram in identifying resting pulmonary artery pressure. Because different factors, such as patient size impacts the size of the pulmonary artery and the AO value equally, Devaraj et al. proposed that the ratio of the MPA to the diameter of the AO is an accurate marker of mPAP [22].

We found a correlation between the RPA and LPA and mean pulmonary arterial pressure (mPAP) to be very weak (r = 0.155, p-value = 0.050, and r = 0.138 respectively). It was not statistically significant in the case of the LPA. The research by Terpenning et al. showed that the addition of RPA and LPA measurements does not increase PH diagnosis accuracy [26]. Essam et al. also correlated the RPA and LPA with mPAP; there was no correlation between LPA and mPAP. On the contrary to our results, RPA showed a strong correlation with mPAP [16].

Our study revealed a weak correlation between vascular measures and mPAP. The studies mentioned above also show low-to-moderate correlations which indicate CT parameters limited power to predict mPAP, reflecting the limited ability of CT parameters to screen PH patients. In the recent pandemic of COVID-19, the physicians’ request for chest CT has increased. Now, PH is also a typical consequence of COVID-19 illness, occurring even in moderate cases following recovery [27]. Even though the CT chest has the limited ability it can be helpful in these cases.

The MPA diameter cut-off values range from 25 to 33.2 mm across different studies [5]. In our population, it was 26 mm with a sensitivity and specificity of 0.608 and 0.53. The cut-off value for PH for MPA/AO ratio was 0.88. This result was close to Chan et al. with a cut-off value of 0.84 [28]. However, in other studies, the cut-off value was more than 1 [18, 23, 25, 29].

Our study has a few limitations: first, we correlated CT parameters of PH with echocardiography rather than gold-standard right heart catheterization, which is an invasive technique and not done on a routine basis. Second, with underlying pulmonary illness, the population was not homogeneous. CT and echocardiography had a maximum gap of one month in the included data. However, ideally, it should be done on the same day [18].

## Conclusions

Although right heart catheterization is the gold standard for measuring PAP, it is an invasive and expensive procedure. As a result, the noninvasive mPAP estimate with echocardiography is particularly appealing. Even though CT parameters of PH correlate with mPAP when measured with echocardiography, the assessment exclusively on CT chest scans might not be reliable enough for clinical application. Furthermore, the cut-off values of CT for PH should not be generalized and taken separately for different ethnicities. Future research should emphasize the role of CT in PH in particular disease subtypes and other ethnicities.
